# Efficiencies and Work Losses for Cycles Interacting with Reservoirs of Apparent Negative Temperatures

**DOI:** 10.3390/e21080749

**Published:** 2019-07-31

**Authors:** Henning Struchtrup

**Affiliations:** Mechanical Engineering, University of Victoria, Victoria, BC V8W 2Y2, Canada; struchtr@uvic.ca

**Keywords:** negative thermodynamic temperature, thermal efficiency, energy storage

## Abstract

Inverted quantum states of apparent negative temperature store the work required for their creation [Struchtrup. *Phys. Rev. Lett.* **2018**, *120*, 250602]. Thermodynamic cycles operating between a classical reservoir and an inverted state reservoir seem to have thermal efficiencies at or even above unity. These high efficiencies result from inappropriate definition adopted from classical heat engines. A properly defined efficiency compares the work produced in the cycle to the work expended in creating the reservoir. Due to work loss to irreversible processes, this work storage based efficiency always has values below unity.

## 1. Introduction

The forced inversion of states in certain quantum systems leads to states that are often described by negative thermodynamic temperatures [1,2,3,4,5,6,7,8]. Recently, building on previous research [9,10,11], we have argued that these states cannot be considered as stable thermodynamic states, and hence should not be associated with a thermodynamic temperature [12]. The instability shows in that an inverted system which is brought into contact with a conventional system will fall into an equilibrium with that system at positive temperatures, independent of the size of the two systems [10]. Nevertheless, invertible systems among each other can equilibrate at inverted states with an equal negative thermodynamic temperature [12]. Accordingly, we discuss systems of *temperature-unstable states* and of *apparent* negative temperatures.

Inverted states at apparent negative temperatures can only be created by work, where the reversible work required for their creation is stored as energy of the inverted state, just like in a battery or mechanical spring. This work can, in principle, be recovered directly by re-inversion to a conventional state with positive temperature [12].

Classical heat engines operate between two reservoirs at different temperatures, where the desire to equilibrate the temperature provides the thermodynamic driving force [13]. In contrast, an engine in contact with a single reservoir at an apparent negative temperature can produce work [2,12]. This behavior of inverted systems does not contradict the second law of thermodynamics, which forbids to produce work from a single reservoir, since a reservoir at apparent negative temperature is not in a stable equilibrium state: The driving force for the work generation is the deviation from a stable thermodynamic state [12].

In the field of quantum thermodynamics, equilibrium states are characterized by the notion of “passivity”, which means that no work can be produced from these states [14]. In this terminology, inverted states are active—work can be produced—and not in equilibrium.

In our previous discussion, we considered a small system that undergoes adiabatic processes between stable states at positive temperatures and temperature-unstable inverted states at apparent negative temperatures [12]. It became clear that all work that can be produced from such a system in an inverted state if it is equal or less than the work required to create the inverted state.

For additional insight into inverted states as storage systems, we now consider thermodynamic engines operating between a classical thermal reservoir, the freely available environment at temperature $T_{0}$, and a reservoir of inverted states. Here, “freely available” implies that no cost is associated with drawing heat from, or dumping heat into the environment at temperature $T_{0}$.

In the Kelvin–Planck formulation, the second law states that heat cannot be fully converted to work [12,13], hence thermal efficiency is always below unity. As discussed first by Ramsey [2], the classical Kelvin–Planck formulation is not valid for inverted states, which can produce work directly. Hence, the classical definition of thermal efficiency—with an expected value below unity—loses its meaning.

Several authors have discussed cycle efficiencies for inverted state reservoirs [2,4,6,15,16]. In [6] the authors point out that “negative temperature heat baths do not occur naturally: they have to be prepared. If the total heat and work budget include the preparation of the negative-temperature heat bath from a system at positive temperature, then it turns out that it is still not possible to run an engine sustainably by extracting heat from a single reservoir. Negative temperatures in real-world laboratory settings do not imply perpetual motion.”

In the spirit of this statement, we consider two simple cycles that draw energy from the inverted reservoir and provide work, with efficiency measures equal to unity or above, depending on the definition. For both cycles, the detailed analysis based on the first and second law of thermodynamics shows that theses cycles reduce the work potential of the reservoir (which for the calculations must be considered as finite, but much larger than the working system) by an amount that is larger than the work produced by the cycle. Hence, only part of the work required to produce the reservoir is recovered through the engine, and there is no contradiction to thermodynamic principles.

## 2. Thermodynamics of Inverted States

To proceed, as in [12], we consider the simplest model possible, where the invertible quantum systems have a single degree of freedom, the occupation number of quantum elements which determines the system’s energy E=ne, where *n* is number of moles of quantum elements in the system, and *e* is the mole-specific energy. There are no other parameters to describe the system or all other parameters remain frozen throughout all processes.

The entropy-energy relation depends on the definition of entropy in statistical mechanics, where the Boltzmann definition leads to apparent negative temperatures, but the Gibbs definition does not [4,6,7,8]. The following discussion relies on the Boltzmann definition of entropy.

**Figure 1:**
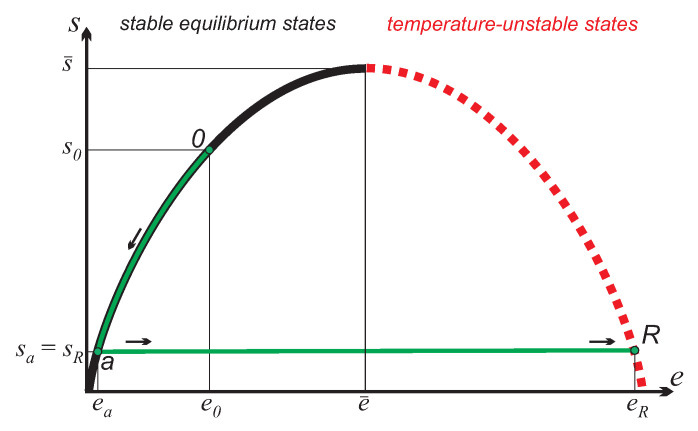
Entropy-energy relation $s\left(e\right)$. States on the ascending part of the curve are stable equilibrium states (black), states on the descending part are temperature-unstable states (red dashes). The process 0-*a*-*R* is the (reversible) charging from equilibrium environmental state 0 to inverted state *R*.

Bounded energy of quantum states and random energy exchange between quantum elements [2,3] lead to a relation between system entropy S=ns and system energy given by a curve $s\left(e\right)$ which first increases to a maximum, and then decreases, see Figure 1. For simplicity we shall assume the symmetry of the curve relative to the maximum $\left\{\bar{e},\bar{s}\right\}$, so that $s\left(\bar{e}+\Delta e\right)=s\left(\bar{e}-\Delta e\right)$ for all accessible $\bar{e}\pm \Delta e$; this assumption agrees with models discussed in the literature [2,6,11].

Left of the maximum, the curve describes conventional stable states at positive temperatures:

(1) $$T={\left[\frac{de}{ds}\right]}_{e<\bar{e}}>0\;,$$

while the states right of the maximum are temperature-unstable states [12] with an apparent negative temperature:

(2) $$\theta ={\left[\frac{de}{ds}\right]}_{e>\bar{e}}<0.$$

Note that states of apparent negative temperature appear to be hotter than conventional states at positive temperature [2,5,12].

For the discussion, we only require the general form of the curve $s\left(e\right)$, and the first and second law of thermodynamics which we write as [13]:

(3) $$\frac{dE}{dt}=\dot{Q}-\dot{W}\;\;\;\;\;,\;\;\;\;\frac{dS}{dt}-\frac{\dot{Q}}{T_{0}}={\dot{S}}_{\mathrm{gen}}\geq 0.$$

Here, $\dot{Q}$ is the heat transfer rate (positive for heat added to the system), $\dot{W}$ is the power (positive for work done by the system), and ${\dot{S}}_{\mathrm{gen}}$ is the entropy generation rate, which is positive in non-equilibrium, and vanishes in equilibrium. All processes to be considered will either be equilibration between two invertible systems that are isolated from their surroundings (no external heat transfer, $\dot{Q}=0$) or involve heat exchange only with the freely available environment at temperature $T_{0}>0$.

Next, we consider the creation of an inverted reservoir of *N* quantum elements from the ground state at temperature $T_{0}$ (state 0 in Figure 1). As discussed in [12,17], inverted states can only be produced by “jumping” from the stable to the unstable branch of the $s\left(e\right)$ curve. Specifically, we first cool the reservoir at a conventional temperature from state 0 to a state *a* by means of a refrigerator that removes the heat $Q_{0a}=\int_{0}^{a}\dot{Q}dt<0$ from the system, and dumps heat into the free environment at $T_{0}$. Then we perform the adiabatic reversible state inversion from state *a* to state *R*, where $Q_{aR}=0$. Integration of the thermodynamic laws (3) along the path 0-*a*-*R* yields (note that $s_{a}=s_{R}$):

(4) $$\begin{array}{ccc} N\left(e_{R}-e_{0}\right) & = & Q_{0a}-W_{0aR}\;\;, \end{array}$$

(5) $$\begin{array}{ccc} N\left(s_{R}-s_{0}\right)-\frac{Q_{0a}}{T_{0}} & = & S_{\mathrm{gen}}^{0aR}\geq 0. \end{array}$$

Elimination of the heat $Q_{0a}$ transferred into the free environment yields the work requirement for establishing the reservoir as:

(6) $$W_{0aR}=-N\left(e_{R}-e_{0}-T_{0}\left(s_{R}-s_{0}\right)\right)-T_{0}S_{\mathrm{gen}}^{0aR}<0.$$

Any irreversibility $S_{\mathrm{gen}}^{0aR}>0$ in the process increases the absolute work requirement. Returning along the same path *R*-*a*-0, in an irreversible process, yields the work:

(7) $$W_{Ra0}=N\left(e_{R}-e_{0}-T_{0}\left(s_{R}-s_{0}\right)\right)-T_{0}S_{\mathrm{gen}}^{Ra0}>0\;,$$

where irreversibilities $S_{\mathrm{gen}}^{Ra0}>0$ reduce the amount of work retrieved.

The reversible work $W^{0}$ stored in the reservoir relative to the free environment is defined as the amount of work that could be retrieved from returning the reservoir to an environmental state in a fully reversible process,

(8) $$W_{N,R}^{0}=N\left(e_{R}-e_{0}-T_{0}\left(s_{R}-s_{0}\right)\right)>0.$$

Note that some of the reversible work is related to inverted states, and some to temperature difference to the free environment.

## 3. Retrieval of Stored Work

### 3.1. Direct Retrieval

The easiest way to retrieve a smaller portion of the work $W_{N,R}^{0}$ stored in the reservoir is to take a small amount *n* of reservoir material and return it in a controlled process along the path *R*-*a*-0 to the environmental state. In an ideal scenario, i.e., reversible case, this yields the reversible work:

(9) $$W_{n,R}^{0}=n\left(e_{R}-e_{0}-T_{0}\left(s_{R}-s_{0}\right)\right)\;.$$

Over time, the full work potential of the reservoir can be retrieved by performing the same process on smaller amounts of material, until the reservoir is, bit-by-bit, completely returned to the environmental state.

### 3.2. Retrieval from Single Reservoir

Instead of directly retrieving work from the reservoir material, we are now interested in engines operating in contact with reservoirs. Here, the reservoirs remain intact, but interact with a thermodynamic engine, in which a working substance undergoes a cyclic change of properties. For simplicity we study systems where the working substance is the same as the reservoir material, so that both share their thermodynamic properties.

**Figure 2:**
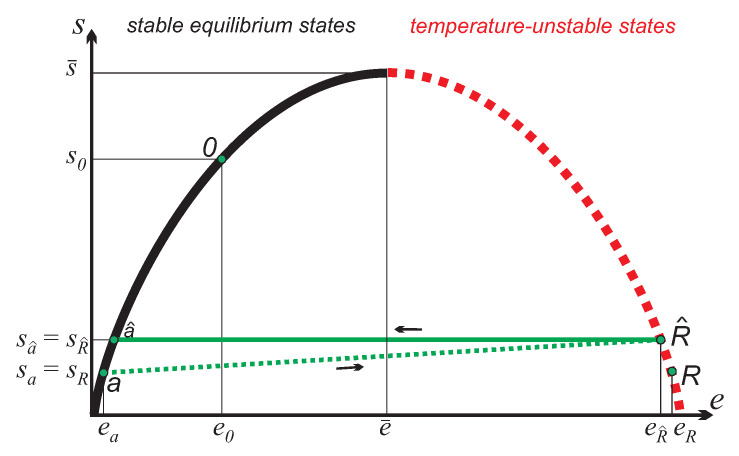
Work and charge process for a *n*-mole working system originally in non-inverted state *a*. The working system assumes an inverted state by irreversible equilibration with the *N*-mole reservoir *R* in process $a\to \hat{R}$. The system returns to a non-inverted state $\hat{a}$ and provides reversible work in the process $\hat{R}\to \hat{a}$. For N≫n the new equilibrium state $\hat{R}$ between the working system and the reservoir is very close to *R*; in the figure the distance is exaggerated for better readability.

Since work can be directly retrieved from inverted states, the simplest cycle possible does not require a second reservoir, but just uses a single inverted reservoir of *N* moles and the working substance of n≪N moles. As a starting point for the process, we consider a reservoir at state $\left\{e_{R},s_{R}\right\}$ and the working substance at state $\left\{e_{a},s_{a}\right\}$, where $s_{a}=s_{R}$. The cycle consists of the following two processes, as depicted in Figure 2:

*a*-$\hat{R}$, charge: Working substance (state *a*) and reservoir (state *R*) equilibrate irreversibly to the common equilibrium state $\hat{R}$ while adiabatically isolated from their surroundings. The first law (3)${}_{1}$ reduces to:

(10) $$\left(n+N\right)e_{\hat{R}}=ne_{a}+Ne_{R}\;,$$

so that the the energy transferred from reservoir to the working substance is:

(11) $$Q_{a\hat{R}}=n\left(e_{\hat{R}}-e_{a}\right)=N\left(e_{R}-e_{\hat{R}}\right)>0.$$

With $s_{a}=s_{R}$, the second law (3)${}_{2}$ reduces to:

(12) $$S_{\mathrm{gen}}^{a\hat{R}}=\left(n+N\right)\left(s_{\hat{R}}-s_{R}\right)>0\;.$$

$\hat{R}$-$\hat{a}$, work production: The working substance returns to non-inverted state $\hat{a}$, in an adiabatic reversible process, where $s_{\hat{a}}=s_{\hat{R}}$ as indicated in Figure 2. The first law (3)${}_{1}$ gives the work produced as:

(13) $$W_{\hat{R}\hat{a}}=n\left(e_{\hat{R}}-e_{\hat{a}}\right)>0\;,$$

and the second law (3)${}_{2}$ simply states isentropicity, $s_{\hat{a}}=s_{\hat{R}}$.

Accordingly, the endpoint $\hat{R}$ of the equilibration process has slightly larger entropy and slightly smaller energy than the initial reservoir state R. As the cycle is repeated, overtime the reservoir state moves up on the curve, towards larger entropies and lower energies. If the reservoir is much larger than the working system, N≫n, the final state of the cycle almost coincides with the initial state. However, the small difference between the two states *R* and $\hat{R}$ will be important for a full understanding of the process.

**Figure 3:**
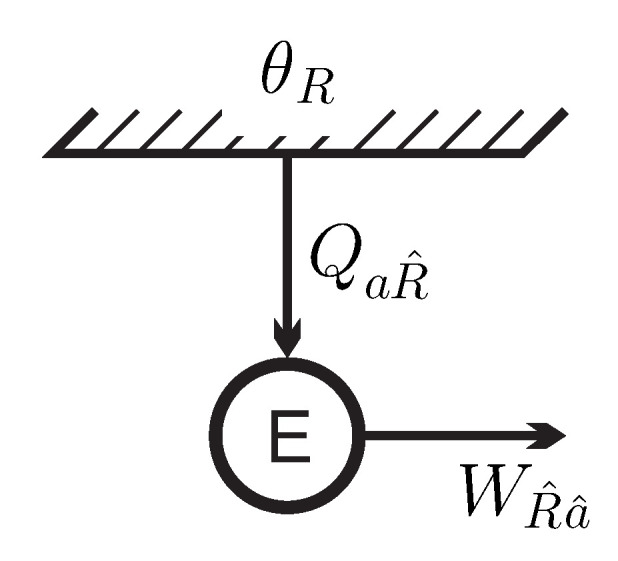
Energy flows for the simple back-forth process producing the work $W_{\hat{R}\hat{a}}$ from the energy $Q_{a\hat{R}}$ drawn from a reservoir at apparent negative temperature $\theta_{R}$.

Figure 3 shows the energy flows for the engine *E* in contact with the inverted reservoir at apparent negative temperature $\theta_{R}$. As discussed before [12], in the present interpretation this process does not contradict the Kelvin–Planck statement, since the reservoir is not in a stable equilibrium state.

Following usual definitions from the thermodynamics of engines [13], the energy efficiency of this simple back and forth (b-f) process is:

(14) $$\eta_{\text{b-f}}=\frac{\mathrm{work}\;\mathrm{produced}}{\mathrm{energy}\;\mathrm{in}}=\frac{W_{\hat{R}\hat{a}}}{Q_{a\hat{R}}}=\frac{e_{\hat{R}}-e_{\hat{a}}}{e_{\hat{R}}-e_{a}}≲1.$$

In this definition, the energy drawn from the reservoir is considered as the expense incurred, while the work produced is considered as the gain. Due to the (very small) difference between start and end state, the resulting efficiency is just a bit below unity, reaching unity for N/n→∞.

A proper efficiency measure should be useful for evaluating the quality of the processes in the engine. The value found here, $\eta_{\text{b-f}}≲1$, is simply reflecting conservation of energy for the cycle, and does not give useful information on the quality of the process.

An efficiency of (almost) unity seemingly contradicts classical thermodynamic statements on heat engine efficiency [13], but, of course, heat engine efficiency is based on the discussion of thermal reservoirs, while the reservoir of inverted states is better seen as a work reservoir that stores the work required for its creation [12], and this work should be seen as the associated cost.

Accordingly, one should not just ask for the energy transferred from the reservoir, but also for the depletion of the reservoir’s work potential. Since we study a process that is always detached from the free environment, the work potential of the reservoir in the original state *R* and the new state $\hat{R}$ is given by the work that can be obtained from adiabatic reversible re-inversion of the reservoir to states *a* or $\hat{a}$, respectively. That is,

(15) $$W_{N,R}=N\left(e_{R}-e_{a}\right)\;\;\;,\;\;\;W_{N,\hat{R}}=N\left(e_{\hat{R}}-e_{\hat{a}}\right)\;$$

Thus, the change of the reservoir’s work potential for one cycle is the difference:

(16) $$\Delta W_{N,R\hat{R}}=W_{N,R}-W_{N,\hat{R}}=N\left(e_{R}-e_{a}-e_{\hat{R}}+e_{\hat{a}}\right).$$

Since N≫n, we can use linear approximations, and find with Equation (1),

(17) $$e_{\hat{a}}-e_{a}≃{\left[\frac{de}{ds}\right]}_{a}\left(s_{\hat{a}}-s_{a}\right)=T_{a}\left(s_{\hat{a}}-s_{a}\right)\;,$$

and, with $s_{\hat{a}}=s_{\hat{R}}$, $s_{a}=s_{R}$, and Equation (2),

(18) $$s_{\hat{a}}-s_{a}=s_{\hat{R}}-s_{R}≃{\left[\frac{ds}{de}\right]}_{R}\left(e_{\hat{R}}-e_{R}\right)=\frac{e_{\hat{R}}-e_{R}}{\theta_{R}}\;.$$

Due to the assumed symmetry of the curve, we have ${\left[\frac{de}{ds}\right]}_{a}{\left[\frac{ds}{de}\right]}_{R}=T_{a}/\theta_{R}=-1$, so that, combining the above with Equation (11), we find the change in work potential as:

(19) $$\Delta W_{N,R\hat{R}}=2n\left(e_{\hat{R}}-e_{a}\right)=2Q_{a\hat{R}}\;.$$

Hence, the change of the reservoir’s work potential is equal to twice the energy transferred from the reservoir to the working substance.

From an engineering perspective, the expense related to the cycle is the work for the creation of the inverted reservoir. Thus, a better measure for the efficiency is the relative amount of work potential used,

(20) $$\eta_{W}=\frac{\mathrm{work}\;\mathrm{produced}}{\mathrm{work}\;\mathrm{potential}\;\mathrm{used}}=\frac{W_{\hat{R}\hat{a}}}{\Delta W_{N,R\hat{R}}}≲0.5.$$

Only half of the change in work potential is realized in the cyclic back-and-forth process, while the other half is lost in the irreversible equilibration process *a*-$\hat{R}$. Indeed, the work loss to irreversibilities is related to the entropy generation (12) as (for N≫n):

(21) $$W_{\mathrm{loss}}=\Delta W_{N,R\hat{R}}-W_{\hat{R}\hat{a}}=T_{a}S_{\mathrm{gen}}^{a\hat{R}}\;.$$

The efficiency $\eta_{W}$ compares actual work produced to work potential consumed and hence belongs to the class of “thermodynamic efficiencies” [18] or “second law efficiencies” [13]. Unambiguously, a thermodynamic efficiency is always below unity, unless the process is fully reversible, and is considered to be a better measure for the quality of a process than an energy efficiency such as $\eta_{\text{b-f}}$ [18].

However, a better interpretation of the efficiency $\eta_{W}$ is as the round trip efficiency of a storage system, where the work retrieved is compared to the work for charging (the change in work potential).

### 3.3. “Otto Engine”

Next, inspired by [19], we consider a process similar to the classical Otto cycle, where heat is exchanged with two thermal reservoirs at different temperatures. The Otto cycle consists of the following four processes [13]: (i) Work production in adiabatic expansion, (ii) isochoric heat exchange with free environment (no work), (iii) work input for adiabatic compression, and (iv) isochoric heat exchange within a high temperature environment (no work).

**Figure 4:**
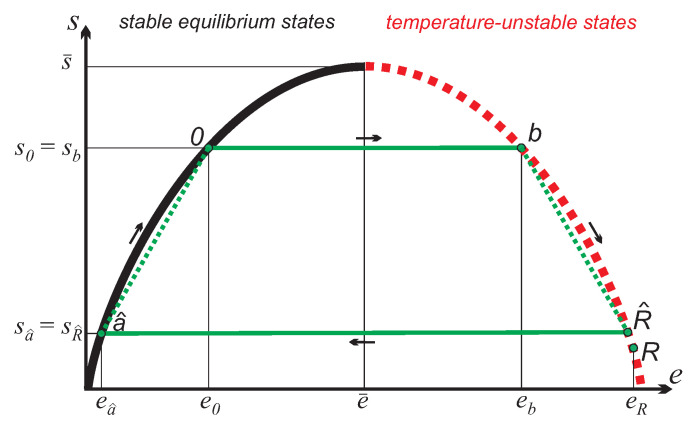
“Otto” cycle between a inverted work reservoir at state *R* and a free environment at state 0. Processes $b\to \hat{R}$ and $\hat{a}\to 0$ are irrversible equilibration processes between the system and the reservoirs at states 0 and *R*, respectively. For N≫n, the endpoint $\hat{R}$ almost coincides with starting point *R*, the distance is exaggerated for better readability of the figure.

The cycle to be studied, shown in Figure 4, similarly involves two adiabatic work processes, and two irreversible exchange processes with the reservoirs without work. For the same working substance and reservoir as before, the four processes are as follows (symmetry of the entropy curve used for some results):

0-*b*: Adiabatic reversible state inversion; work input:

(22) $$W_{0b}=n\left(e_{0}-e_{b}\right)<0\;.$$

*b*-$\hat{R}$: Irreversible equilibration with inverted reservoir to common equilibrium $\hat{R}$; no work, energy transfer:

(23) $$Q_{bR}=n\left(e_{\hat{R}}-e_{b}\right)=N\left(e_{R}-e_{\hat{R}}\right)>0\;.$$

$\hat{R}$-$\hat{a}$: Adiabatic reversible return from inverted states; work produced:

(24) $$W_{\hat{R}\hat{a}}=n\left(e_{\hat{R}}-e_{\hat{a}}\right)>0\;.$$

$\hat{a}$-0: Irreversible equilibration with free environment at $T_{0}$; no work, energy transfer:

(25) $$Q_{\hat{a}0}=n\left(e_{0}-e_{\hat{a}}\right)>0\;.$$

Due to the assumed symmetry of the entropy curve, the net work produced by the cycle is:

(26) $$W_{\mathrm{Otto}}=W_{\hat{R}\hat{a}}+W_{0b}=n\left(e_{\hat{R}}-e_{\hat{a}}+e_{0}-e_{b}\right)=2n\left(e_{\hat{R}}-e_{b}\right).$$

**Figure 5:**
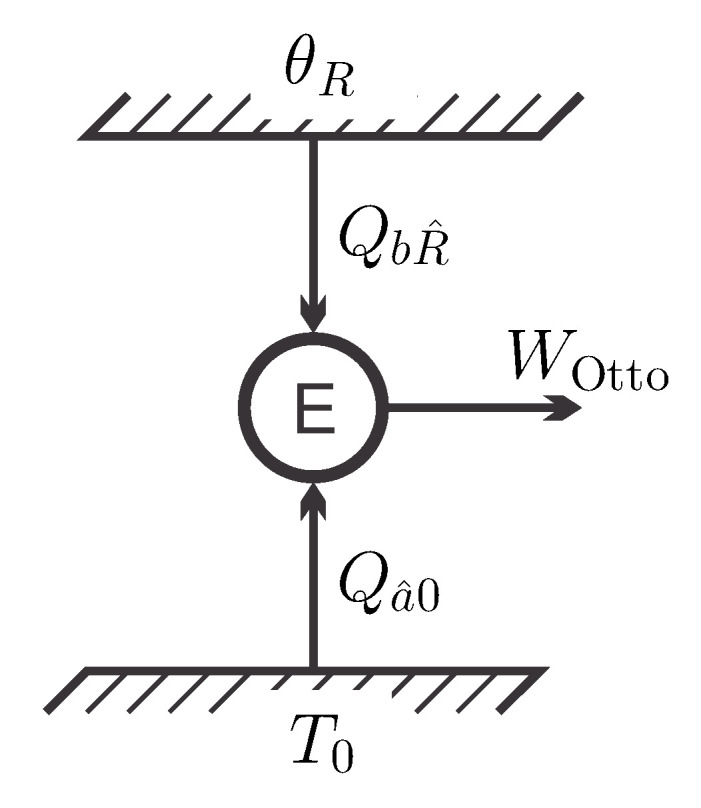
Energy flow diagram for the “Otto” cycle between a inverted work reservoir at state *R* and a free environment at state 0. The engine draws heat from both reservoirs to produce work.

Figure 5 shows the energy flows for this process and again we consider the question of meaningful definitions for the efficiency of the cycle.

A natural choice for the definition of energy efficiency is to compare the work produced to the energy drawn into the cycle [7], so that:

(27) $$\eta_{\mathrm{Otto}}=\frac{W_{\mathrm{Otto}}}{Q_{b\hat{R}}+Q_{\hat{a}0}}=\frac{n\left(e_{\hat{R}}-e_{\hat{a}}+e_{0}-e_{b}\right)}{n\left(e_{\hat{R}}-e_{b}+e_{0}-e_{\hat{a}}\right)}=1\;.$$

This gives an efficiency at unity, just as for the simple back-and-forth cycle, see Equation (14). Again, this is simply a reflection of energy conservation for the cycle, with energy drawn from the reservoirs fully converted to work.

Both cycles draw energy from the same inverted reservoir and have energy efficiency of unity. That is, from the viewpoint on energy efficiency, one cannot decide which of the processes is the better choice for the exploitation of the reservoir.

As before, the storage efficiency, which compares work produced to change of work potential, provides a clearer picture and deeper insight. The work potential of the inverted reservoir relative to the free environment is given by Equation (8), hence the change of work potential between states *R* and $\hat{R}$, after one cycle of the “Otto” engine, is:

(28) $$\Delta W_{N,R\hat{R}}=W_{N,R}-W_{N,\hat{R}}=N\left(e_{R}-e_{\hat{R}}-T_{0}\left(s_{R}-s_{\hat{R}}\right)\right).$$

Insertion of Equations (18) and (23) yields the change of work potential due to one run of the cycle as:

(29) $$\Delta W_{N,R\hat{R}}=n\left(e_{\hat{R}}-e_{b}\right)\left(1+\frac{T_{0}}{T_{a}}\right)\;,$$

where we used ${\left[-\frac{ds}{de}\right]}_{R}=-1/\theta_{R}={\left[\frac{ds}{de}\right]}_{a}=1/T_{a}$, due to the assumed symmetry of the curve s(e).

Comparing work produced to work potential used gives:

(30) $$\eta_{W}=\frac{W_{\mathrm{Otto}}}{\Delta W_{N,R\hat{R}}}=\frac{2}{1+\frac{T_{0}}{T_{a}}}<1\;.$$

Note that in the limit $T_{a}\to T_{0}$ the storage efficiency $\eta_{W}$ approaches unity, while the net work of the cycle vanishes.

As expected, the cycle does not fully realize the work potential drawn from the reservoir. The difference is due to generation of entropy in the energy transfer processes *b*-$\hat{R}$ and $\hat{a}$-0 (equilibration between working substance and reservoirs, without work). A short calculation yields the overall entropy generation as:

(31) $$S_{\mathrm{gen}}^{\mathrm{Otto}}=n\left(e_{\hat{R}}-e_{b}\right)\left(\frac{1}{T_{a}}-\frac{1}{T_{0}}\right)>0\;,$$

which is, as ususal in thermodynamics [13], related to the work lost through the environmental temperature $T_{0}$ as:

(32) $$W_{\mathrm{loss}}=\Delta W_{N,R\hat{R}}-W_{\mathrm{Otto}}=T_{0}S_{\mathrm{gen}}^{\mathrm{Otto}}.$$

In summary, we state that energy based efficiencies of unity for cycles in contact with an inverted reservoir only reflect the conservation of energy and do not give insight into cycle performance. Indeed, based on this information, one would have difficulty choosing.

However, if one considers the inverted reservoir as a storage system, one can compare the work produced to the change of work potential of the reservoir, as in Equations (20) and (30). Indeed, comparing these values shows that the Otto engine performs better than the simple back-forth process, as long as $T_{0}/T_{a}<3$.

## 4. Super-Efficient Engines?

If one considers the standard Carnot efficiency $\eta_{C}=1-\frac{T_{L}}{\theta_{H}}$ between a conventional reservoir at temperature $T_{L}>0$ and an inverted reservoir at (apparent) negative temperature $\theta_{H}<0$, one finds (apparent) efficiencies above unity.

For a better understanding of why these efficiencies above unity could arise, we look at this for the “Otto” engine above, and consider a cost based definition of efficiency, where the exchange $Q_{a0}$ with the environment at $T_{0}$ is considered to be free, and only the energy $Q_{bR}$ drawn from the inverted reservoir is considered as an expense.

Indeed, in engineering thermodynamics, the heat exchanged with the free environment in either direction is typically not considered a cost [13]. For example, a heat pump receiving the work $\left|W\right|$, draws heat $Q_{0}$ from the free environment at $T_{0}$, and provides the heat $Q_{H}=\left|W\right|+\left|Q_{0}\right|$ at some elevated temperature $T_{H}>T_{0}$. Its coefficent of performance considers only the work input as a cost, ${\mathrm{COP}}_{\mathrm{HP}}=\frac{\left|Q_{H}\right|}{\left|W\right|}=1+\frac{\left|Q_{0}\right|}{\left|W\right|}\geq 1$ [13].

Based on this consideration, one might define a cost-based coefficient of performance as:

(33) $${\mathrm{COP}}_{\mathrm{Otto}}=\frac{W_{\mathrm{Otto}}}{Q_{b\hat{R}}}=\frac{2n\left(e_{\hat{R}}-e_{b}\right)}{n\left(e_{\hat{R}}-e_{b}\right)}=2.$$

Since a proper efficiency assumes values in the interval (0,1), it is certainly better to not call this ratio an efficiency.

Similar to the COP of a heat pump, the COP of Equation (33) considers the heat $Q_{a0}$ drawn from the environment at $T_{0}$ as free. It associates a cost only with the energy drawn from the inverted reservor, $Q_{b\hat{R}}$. This leads to an efficiency measure above unity. There is no contradiction to thermodynamic principles, only the question of how useful such a definition might actually be.

Efficiency measures are not naturally given, but defined in order to give meaningful insights into the behavior of (thermodynamic) systems. In our opinion, both measures, $\eta_{\mathrm{Otto}}$ and ${\mathrm{COP}}_{\mathrm{Otto}}$, fail to be meaningful, since they ignore that the creation of the inverted reservoir implies a cost. That is, in their definitions the reservoir is treated as a resource and not as a storage medium, thus the cost for its creation is not properly considered.

In other words, the problem of definition (33) is not that the heat drawn from the environment at $T_{0}$ is not considered, but rather that a definition that is useful for thermal reservoirs (which are in equilibrium states) is transferred to the evaluation of a storage system which is not in a classical equilibrium state.

The proper measure to evaluate energy storage systems is the roundtrip efficiency, that is comparing energy retrieved to energy charge, and this is just what the efficienies $\eta_{W}$ from Equations (20) and (30) do.

## 5. Conclusions

The above analysis confirmed that cycles drawing energy from reservoirs in inverted states do not contradict the laws of thermodynamics [6]. In particular, any amount of work produced by the cycles is equal or less than the amount of work required to generate the reservoir. Moreover, work lost to irreversibilities is proportional to the generation of entropy, with a (positive) temperature as a factor, in agreement with classical engineering thermodynamics [13].

A proper efficiency measure aims to compare the gain produced to the expense incurred. For a classical heat engine, the expense is due to the amount of fuel required to maintain the temperature of the hot reservoir, where the fuel’s heat of reaction equals the amount of heat drawn by the engine.

An inverted reservoir can only be created by work input, and for a meaningful definition of efficiency this work should be considered as the expense, as in $\eta_{W}$ Equations (20) and (30).

In short, reservoirs in inverted states at apparent negative temperatures must be considered as energy storage, and not as heat reservoirs, since they can only be produced by work input. Accordingly, their thermodynamic behavior differs from that of classical thermal reservoirs, not least in that the work can be produced from a single reservoir. Meaningful efficiency measures should take this difference into account and should not just be transferred from the discussion of thermal systems.

Heat reservoirs can be maintained continuously by, e.g., combustion of fuel, so that a heat engine continuously produces work from the fuel. An inverted state reservoir can only be maintained at steady state by work input and hence requires the very resource that is drawn by the thermodynamic cycle.

Since inverted states are difficult to produce and maintain, one would expect to have smaller systems available rather than large reservoirs. Since work is directly available from the inverted states, it is thermodynamically preferable to obtain work from the re-inversion process and not from cycles, which are charged by interaction with inverted state reservoirs. Due to the inherent irreversibilities, the charging process reduces the (possible) work output.

In short, thermodynamic cycles in interaction with reservoirs at apparent negative thermodynamic temperatures are interesting only as a concept. Should inverted states ever play a role as energy storage systems, one should aim to charge and discharge the systems directly.
